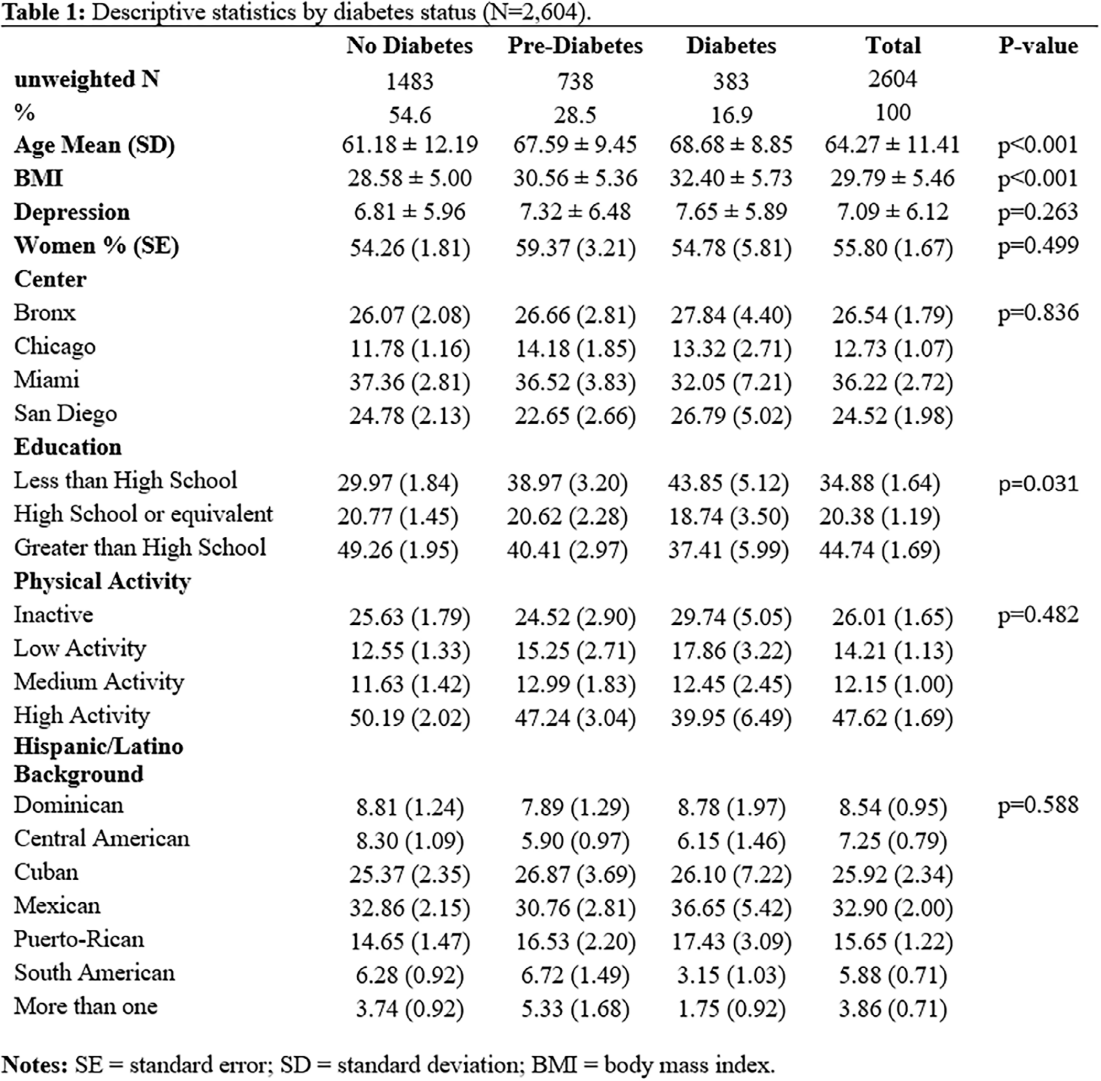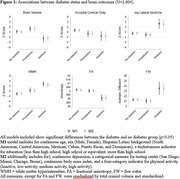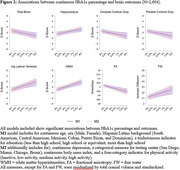# Diabetes and brain MRI measures: Results from SOL‐INCA MRI study

**DOI:** 10.1002/alz.089616

**Published:** 2025-01-09

**Authors:** Kevin A Gonzalez, Wassim Tarraf, Sarah Banks, Katherine J. Bangen, Judy Pa, Linda C Gallo, Ariana M Stickel, Paola Filigrana, Carmen R Isasi, Martha L Daviglus, Fernando Daniel Testai, Charles Decarli, Hector M Gonzalez

**Affiliations:** ^1^ University of California San Diego, La Jolla, CA USA; ^2^ University of California, San Diego, La Jolla, CA USA; ^3^ Wayne State University, Detroit, MI USA; ^4^ UC San Diego, La Jolla, CA USA; ^5^ VA San Diego Healthcare System, San Diego, CA USA; ^6^ Alzheimer's Disease Cooperative Study (ADCS), University of California, San Diego, La Jolla, CA USA; ^7^ Neurosciences Graduate Program, University of California, San Diego, La Jolla, CA USA; ^8^ UC San Diego Health, La Jolla, CA USA; ^9^ San Diego State University, San Diego, CA USA; ^10^ Albert Einstein College of Medicine, Bronx, NY USA; ^11^ University of Illinois at Chicago, Chicago, IL USA; ^12^ UIC, Chicago, IL USA; ^13^ University of California, Davis School of Medicine, Sacramento, CA USA; ^14^ University of California San Diego, San Diego, CA USA

## Abstract

**Background:**

Hispanic/Latino communities in the US are rapidly growing and aging and are at two‐fold risk of Alzheimer’s Disease and Related Dementia’s (ADRD) compared to non‐Hispanic Whites. This additional risk could be, in part, due to increased risk of cardiovascular disease. Hispanics/Latinos also have higher rate of diabetes compared to non‐Hispanic Whites and nearly 2 out of 5 individuals with diabetes go undiagnosed. While diabetes has been linked to white matter hyperintensities (WMHs), less is known about the magnitude in diverse Hispanics/Latinos and even fewer studies have considered links to other neurobiological endpoints in this population. This work aims to clarify these associations in a deeply characterized diverse middle‐aged and older Latino cohort.

**Method:**

We used data from the Study of Latinos‐Investigation of Neurocognitive Aging (SOL‐INCA) MRI study which is an ancillary study of the Hispanic Community Health Study/Study of Latinos (HCHS/SOL). The goal of SOL‐INCA MRI is to understand cerebrovascular pathology and ADRD etiology using MRI. A total of N=2400 middle aged and older (50+ years) and N=400 younger (35‐49 years) participants were recruited from the parent HCHS/SOL study. Diabetes was assessed using both continuous HbA1c % and recommended ADA cutoffs to assess prediabetes and diabetes status. Outcomes included structural (i.e., volumes; WMHs, total brain, regional and total gray matter volumes, lateral ventricles, hippocampus) and diffusion MRI (free water=FW, fractional anisotropy=FA). Survey weighted linear models (covariate adjusted by important confounders) were used to test the associations between our exposures and outcomes of interest.

**Result:**

Compared to no diabetes, diabetes was linked to smaller total brain and occipital volumes, larger WMHs, larger lateral ventricles, lower FA, and higher FW (Figure 1). Higher levels of HbA1c % (treated continuously) were additionally associated with smaller occipital and larger hippocampal volumes (Figure 2). These findings were consistent across men and women. We did not find significant differences with the pre‐diabetes group.

**Conclusion:**

Our findings suggest that elevated HbA1c and diabetes status are linked to cerebrovascular pathology and brain atrophy. Findings with hippocampal volume could be due to maladaptive process or hypertrophy. Further research is needed to understand different neurobiological mechanisms in understudied communities.